# Machine learning approaches in identifying factors associated with hypertension and undiagnosed hypertension in adults in rural areas of Bangladesh

**DOI:** 10.1186/s13690-026-01941-z

**Published:** 2026-05-18

**Authors:** Farzana Akhter Bornee, Mohammad Rocky Khan Chowdhury, Md Zahidul Islam, Zarin Raihana, Hasina Akhter Chowdhury, Manzur Kader, Mamunur Rashid

**Affiliations:** 1 Department of Pediatrics, Bangabandhu Sheikh Mujib Medical University, Dhaka, Dhaka Division Bangladesh; 2 Department of Population Science, Jatiya Kabi Kazi Nazrul Islam University, Mymensingh, Mymensingh Division Bangladesh; 3 Department of Epidemiology and Preventive Medicine, School of Public Health and Preventive Medicine, Monash University, Clayton, Melbourne, VIC Australia; 4 Department of Public Health, First Capital University of Bangladesh, Chuadanga, Khulna Division Bangladesh; 5 Institute of Biological Sciences, University of Rajshahi, Rajshahi, Rajshahi Division Bangladesh; 6 Department of Clinical Psychology, Faculty of Biological Sciences, University of Rajshahi, Rajshahi, Rajshahi Division Bangladesh; 7 Department of Medical Science, School of Health and Welfare, Dalarna University, Falun, Sweden; 8 Faculty of Health and Working Life, Unit of Public Health Sciences, University of Gävle, Gävle, Sweden

**Keywords:** Hypertension, Unaware, Machine learning, Rural area, Bangladesh

## Abstract

**Background:**

Hypertension is a major cause of death and disability, and undiagnosed cases are particularly dangerous as they can cause severe damage without timely treatment. The aim of the study was to identify risk factors for hypertension and undiagnosed hypertension in rural areas of Bangladesh using advanced Machine Learning (ML) algorithms.

**Methods:**

This study involved 1,603 respondents, selected through a cross-sectional survey using a multistage cluster random sampling technique. Four ML algorithms, including Gradient Booster (GB), Logistic Regression (LR), Random Forest (RF) and Support Vector Machine (SVM), were used in this study. Risk factors for hypertension and undiagnosed hypertension were identified using the best-performing ML model, selected based on metrics such as accuracy, sensitivity, specificity, precision, F1 score, receiver operating characteristics-area under the curve (ROC-AUC), and calibration plot.

**Results:**

The prevalence of hypertension was 15.5%, slightly higher than the 15.4% for undiagnosed hypertension. In predicting the risk of both hypertension and undiagnosed hypertension, the LR model outperformed other ML models across most evaluation metrics. For hypertension, it achieved higher performance in terms of precision (0.580), F1 score (0.550), ROC-AUC (0.729; 95% CI: 0.677–0.779), and calibration. Similarly, for undiagnosed hypertension, the LR model showed better precision (0.580), ROC-AUC (0.596; 95% CI: 0.537–0.654), and calibration compared to other models. The risk factors for hypertension and undiagnosed hypertension differed notably. Key risk factors for undiagnosed hypertension included being overweight or obese, the absence of chronic diseases or cardiovascular disease (CVD), being male, non-use of tobacco, older age (above 50 years), being currently married, non-smoking status, having diabetes, and having no formal education.

**Conclusion:**

The findings emphasize the urgent need for enhanced national and regional public health initiatives to improve the detection and awareness of hypertension in rural Bangladesh. Further research is important to validate the findings.

**Supplementary Information:**

The online version contains supplementary material available at 10.1186/s13690-026-01941-z.

**Table:** 

Text box 1. Contributions to the literature
• Evidence on factors contributing to undiagnosed hypertension in rural Bangladeshi population remains limited, emphasizing the critical need for sustained and systematic monitoring of non communicable disease trends in rural settings.
• Applications of machine learning (ML) offer strong potential to predict hypertension status and to identify the most influential factors associated with both hypertension and undiagnosed hypertension.
• Factors associated with hypertension and undiagnosed hypertension among rural Bangladeshi populations differ substantially, reflecting variations in health awareness, access to care, and underlying risk profiles.

## Introduction

Hypertension has emerged as a critical global public health concern, affecting approximately one in four adults worldwide [1]. Hypertension and pre-hypertension cause over 8.5 million global deaths per year from stroke, heart disease, vascular, and renal conditions [2, 3]. The growing burden of hypertension is not affecting all regions equally. Low and middle-income countries, especially in South Asia, are seeing a significant rise in hypertension cases [4]. Disturbing trends, especially in countries like Bangladesh, underscore the urgent need for a comprehensive understanding of the risk factors associated with hypertension [5]. In Bangladesh as a developing nation, epidemiological and demographic shifts have contributed to a concerning rise in hypertension rates alongside other non-communicable diseases (NCDs) [6]. In 2018, the prevalence of hypertension among adults exceeds 28% in Bangladesh [7].

In Bangladesh, hypertension affects about 25% of the rural population, where nearly 67% of the country’s people reside [8]. The rural population is facing an increasing incidence of chronic diseases, such as hypertension, which not only affects their health but also threatens their livelihoods [9]. In these communities, the lack of healthcare access, coupled with inadequate media coverage and information, significantly increases the risk of hypertension [10]. Furthermore, the financial strain of managing hypertension, particularly the high out-of-pocket (OOP) expenses, adds another layer of difficulty for rural populations [11]. In Bangladesh, healthcare is primarily financed through OOP payments, unlike developed countries, where government funding and private insurance cover most costs. This reliance on direct household spending places a heavy financial strain on rural families, often forcing them to delay treatment, borrow money, or sell assets. The contrast underscores the lack of financial protection mechanisms in Bangladesh and highlights the vulnerability of rural households to economic hardship when faced with medical expenses [12, 13].

The existing evidence indicates that achieving awareness and control of blood pressure poses challenges in rural areas [14]. While many individuals with hypertension can manage their high blood pressure (BP) through lifestyle modifications, medications, or a combination of both, a significant number of adults are unaware of their condition. Often, symptoms do not manifest until damage has occurred [15]. That gap in hypertension status in rural areas is attributed to broader social and demographic determinants [5]. Research on the prevalence and factors influencing hypertension and undiagnosed hypertension in rural Bangladesh is limited. The national prevalence of hypertension and undiagnosed hypertension was approximately 12.2% and 15.8%, respectively [7]. In a rural district of Bangladesh, it was estimated that over 80% of hypertensive patients were previously unaware [16]. Furthermore, various factors, including social, demographic, and clinical aspects, were identified as associated with undiagnosed hypertension [17].

Most of the previous studies have relied on traditional regression models to identify factors associated with hypertension and undiagnosed hypertension in Bangladesh [7, 16, 18]. While the application of Machine Learning (ML) in predicting hypertension in Bangladesh has been evident [19–21], its use in determining factors associated with hypertension and undiagnosed hypertension, especially in rural communities, remains unexplored. Moreover, evidence suggests that ML methods outperform traditional regression models in prediction and factor selection [22]. Over time, machine learning has become increasingly popular in public health because of growing data availability, advances in computational power, and the need for predictive tools. It is now widely applied in areas such as disease surveillance, risk prediction, health behavior analysis, and resource allocation, reflecting a steady shift toward data-driven decision-making in the field [23, 24].

Given the diverse characteristics of rural communities across different regions within a country, it is essential to conduct more nuanced, community-based studies to capture local health dynamics effectively. Moreover, consistent monitoring of non-communicable disease patterns over time in rural settings is vital, particularly given their worsening trends. Such efforts are increasingly important to inform targeted interventions, allocate resources efficiently, and address the rising burden of hypertension in these often-underserved populations. Therefore, this study aims to identify risk factors for hypertension and undiagnosed hypertension using ML algorithms in rural areas of Bangladesh, thereby enhancing our understanding of these conditions and enabling effective management to improve public health outcomes.

## Materials and methods

### Survey management

The recruitment process for field personnel and the training workshop (S1), the data collection instruments (S2), the pilot survey (S3), and the quality assurance (S4) were presented in the Additional file.

### Sample size calculation/power analysis

The study collected blood pressure (BP) data from village residents and determined the sample size using the formula: n = Zα/2 × p(1 - p) ÷ d². Here, n represents the required sample size, p is the prevalence of hypertension in rural Bangladesh (approximately 27% based on a recent study), and d is the desired accuracy level (set at 3%) (8). With a standard normal deviate (Z1 − α/2) of 1.96 for a 95% confidence level, the initial sample size was calculated to be 841 participants. Adjusting for a design effect of 1.75 to accommodate the multi-stage study design, the final sample size was determined to be1472 [25, 26]. After adjusting the non-response rate, which was around 8.2%, the information was collected from 1603 participants in the actual study (adjusted = 1472/(1-0.082) = 1603).

### Study design and sampling

This was a cross-sectional study conducted from August to November 2021. Geographically, Bangladesh is administratively divided into eight divisions: Barisal, Chittagong, Dhaka, Khulna, Mymensingh, Rajshahi, Rangpur, and Sylhet. The study followed a multi-stage random selection process, beginning with Khulna division. Within Khulna, Jhenaidah district, one of its ten districts, was randomly chosen. From there, Jhenaidah Sadar Upazila, one of the district’s six Upazilas, was selected. The process continued with the selection of Naldanga Union, one of 14 Unions in Jhenaidah Sadar. Participants were drawn from 18 villages in Naldanga Union, and four additional villages were excluded once the target sample size was reached. At least 80 participants were interviewed from each village.

This study utilized the ‘Kish Grid’ method to select households and identify study participants [27]. The ‘Kish Grid’ is a systematic sampling technique used in survey research to ensure representative samples from geographically dispersed populations. It involves listing eligible individuals at an address by age and selecting participants based on the address’s serial number, ensuring equal selection probability within households. This method minimizes bias, ensures randomness, and provides comprehensive population coverage. Effective implementation requires consideration of factors like grid size, resolution, and potential clustering effects.

This method allowed interviewing only one member per selected household. Interviewers began with the household closest to the Union Parishad center and enrolled participants based on inclusion criteria: adults aged 18 and above residing in the household, consenting to participate [28]. Pregnant women, the mentally disabled, and individuals who had surgery within the last three months were excluded to minimize bias. If a household member declined or the household was inaccessible, it was marked as a refusal, and the next household was approached. Proportional representation was ensured by balancing respondents by sex (male and female) and age groups (young adults, adults, middle-aged, and older adults).

### Outcome measures

The study’s outcome variables were hypertension and undiagnosed hypertension. Hypertension was determined based on the BP classification criteria outlined in the Seventh Report of the Joint National Committee (JNC 7), where a systolic blood pressure (SBP) > 140 mmHg and/or diastolic blood pressure (DBP) > 90 mmHg indicated hypertension [29]. Participants were classified as hypertensive if they had a documented diagnosis from a registered medical professional or were taking prescribed antihypertensive medications at the time of data collection [30]. Undiagnosed hypertension referred to participants with an average SBP > 140 mmHg and/or DBP > 90 mmHg who were not undergoing antihypertensive treatment (determined by checking medical records, prescriptions or self-reported) at the time of data collection.

All anthropometric measurements and BP assessments were conducted by a registered medical nurse. Blood pressure was measured using a digital monitor (ELITE YE770A) equipped with a standard-sized cuff. The device was routinely calibrated by inspecting for any damage, connecting it to a certified reference manometer, and verifying accuracy at multiple pressure points (e.g., 50, 100, 150, and 200 mmHg). Readings were required to fall within an acceptable error margin of ± 3 mmHg compared to the reference standard.

Participants were asked to avoid drinking coffee or smoking for at least 30 min before the measurement. They were then instructed to sit in a relaxed position, leaning back with their legs uncrossed and feet flat on the floor, for five minutes. BP was measured on the arm free of excess clothing, positioned at heart level, and the same arm was used for all measurements to ensure consistency. Each participant’s BP was recorded three times, with a 15-minute interval between the first and second, and between the second and third measurements. The mean BP with 95% confidence interval (CI) for each measurement sample was calculated for comparison (Additional file: Supplementary Table 1) The final BP value was calculated as the average of the three readings.

### Independent variables

The study included independent variables derived from findings in the existing published literature [7, 9, 31–33], such as demographic characteristics, which includes gender, age, marital status, educational status, employment status; lifestyle factors, such as smoking history, chewing tobacco; anthropometric characteristics such as, body mass index (BMI) and waist-hip measurement; clinical characteristics such as diabetes, cardiovascular disease (CVD), other chronic diseases, family history of hypertension, presence of anxiety symptoms, and presence of depression symptoms. The operational definitions and measurement scales of the independent variables were provided in Additional file: Supplementary Table 2.

### Statistical analysis

Baseline characteristics were assessed through descriptive analysis. The relationship between the independent and dependent variables was examined using a Chi-square test, with significance set at *p* < 0.05. The first-degree interaction effect was examined among independents variables using logistic regression before final analysis.

### Selection of the best ML algorithm

ML approaches are highly effective in handling complex, non-structured big data from patient databases. They excel at identifying intricate patterns within large datasets containing numerous variables, enabling the exploration of multiple interactions and nonlinear relationships with the outcome, relationships that traditional statistical methods may struggle to explain [34–36]. From a widespread literature search, some ML algorithms were found superior in developing models to predict hypertension, which include Gradient Booster (GB), Logistic Regression (LR), Random Forest (RF) and Support Vector Machine (SVM) [21]. These ML techniques can select variables after model fit [37].

**Fig. 1:**
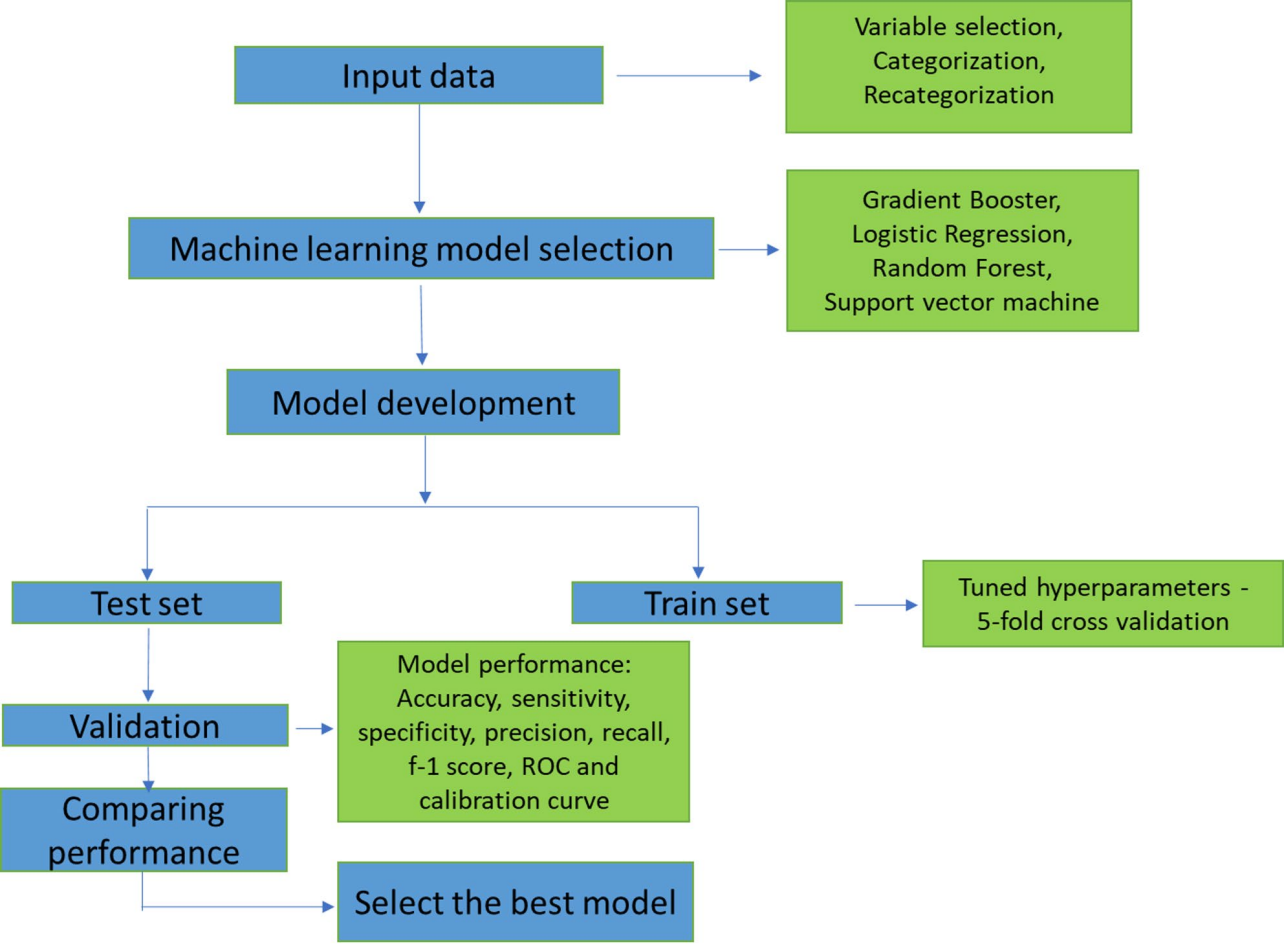
Schematic presentation of selecting the best Machine Learning (ML) model

The ML model was trained on 60% of the dataset (the training dataset), and the remaining 40% was used for validation (the test dataset). The class imbalance of outcome was addressed using the Adaptive Synthetic (ADASYN) resampling technique. Each trained model was optimised with hyperparameter tuning using a 5-fold cross-validation protocol during development (Additional file: Supplementary Table 3). Subsequently, the trained model was validated in 40% validation dataset. The best ML algorithm was selected based on the comparison of performance metrics (accuracy, sensitivity/recall, specificity, precision, F1 score, receiver operating characteristic (ROC) area under the curve (ROC-AUC) curve with 95% CI, and calibration plot (Additional file: Supplementary Table 4) in the validation dataset [38, 39]. A schematic presentation of the best ML model selection is shown in Fig. 1.

### Potential risk factor selection and interpretability in the model

The most significant risk factors for predicting hypertension and undiagnosed hypertension were identified using the SHapley Additive exPlanations (SHAP) technique, developed by Lundberg and Lee [40], which has proven effective in highlighting key risk factors. SHAP is based on game theory principles [41] and local explanation methods [42], and it estimates the contribution of each feature to the model’s overall decision-making process.

Data analysis was carried out using statistical software packages, specifically Stata (version 17), R (latest version), and Python (latest version).

## Results

### Background profile

Approximately 26.7% of the respondents were aged 50 years and older. The gender distribution among respondents was nearly equal, with 51% being male. About 27.4% of respondents reported having no formal education. A smoking habit was present in 19.7% of respondents during the data collection period. Around 56.8% of respondents were classified as overweight or obese. Diabetes affected around 8% of respondents, while 5.4% had some form of CVD. A family history of hypertension was reported by 27.8% of respondents. Symptomatic anxiety was documented in approximately 16% of individuals, and around 18% experienced symptoms of depression. The detailed background profile was presented in Table 1.

**Table 1:** Background characteristics of the study participants

Factors	Frequency	Percentages
*Demographic characteristics*		
Age (in years)		
30 and less	377	23.5
31–50	799	49.8
50 and above	427	26.7
Sex of the respondent		
Male	818	51.0
Female	785	49.0
Educational status		
No formal education	439	27.4
Primary	409	25.5
Secondary	572	35.7
Higher	183	11.4
Employment status		
Employed or self-employed	714	44.5
Housewife	713	44.5
Retired or student	176	11.0
Marital status		
Never married, separated, divorced or widowed	195	12.2
Currently married	1408	87.8
*Lifestyle factors*		
Smoking history		
Never and past smoker	1287	80.3
Current smoker	316	19.7
Chewing tobacco		
Never and past user	1218	76.0
Current user	385	24.0
*Anthropometric characteristics*		
Body mass index		
Normal and underweight	693	43.2
Overweight/pre-obesity	591	36.9
Obese	319	19.9
Waist–hip ratio		
Low	453	28.3
Moderate	313	19.5
High	837	52.2
*Clinical characteristics*		
Diabetes		
No	1474	92.0
Yes	129	8.0
Cardiovascular diseases		
No	1517	94.6
Yes	86	5.4
Other chronic disease		
No	1454	90.7
Yes	149	9.3
Family history of hypertension		
No	1157	72.2
Yes	446	27.8
Presence of anxiety symptoms		
No	1,347	84.0
Yes	256	16.0
Presence of depression symptoms		
No	1314	82.0
Yes	289	18.0

### Prevalence of hypertension and undiagnosed hypertension

The prevalence of hypertension stood at 15.5%, while undiagnosed hypertension was slightly lower at 15.4% in rural areas. Both hypertension and undiagnosed hypertension showed significantly higher rates among individuals aged 50 years and above, with percentages of 28.1% and 19.2%, respectively. Similarly, there was a significant increase in hypertension and undiagnosed hypertension among individuals classified as obese, with proportions of 26.3% and 21.6%, respectively (Table 2).

**Table 2:** Prevalence of hypertension and undiagnosed hypertension

Factors	HTN		Undiagnosed HTN	
	*N* (%)	*p*-value	*N* (%)	*p*-value
Age (in years)				
30 and less	10 (2.7)	< 0.001	41 (10.9)	0.005
31–50	119 (14.9)		124 (15.5)	
50 and above	120 (28.1)		82 (19.2)	
Sex of the respondent				
Male	103 (12.6)	0.001	140 (17.1)	0.053
Female	146 (18.6)		107 (13.6)	
Educational status				
No formal education	89 (20.1)	0.009	76 (17.3)	0.616
Primary	63 (15.4)		62 (15.2)	
Secondary	73 (12.8)		83 (14.5)	
Higher	24 (13.1)		26 (14.2)	
Employment status				
Employed or self-employed	80 (11.2)	< 0.001	115 (16.1)	0.309
Housewife	134 (18.8)		100 (14.0)	
Retired or student	35 (19.9)		32 (18.2)	
Marital status				
Never married, separated, divorced or widowed	32 (16.4)	0.718	22 (11.3)	0.089
Currently married	217 (15.4)		225 (16.0)	
Smoking history				
Never and past smoker	224 (17.4)	< 0.001	207 (16.1)	0.131
Current smoker	25 (7.9)		40 (12.7)	
Chewing tobacco				
Never and past user	185 (15.2)	0.498	198 (16.3)	0.095
Current user	64 (16.6)		49 (12.7)	
Body mass index				
Normal and underweight	62 (9.0)	< 0.001	70 (10.1)	< 0.001
Overweight/pre-obesity	103 (17.4)		108 (18.3)	
Obese	84 (26.3)		69 (21.6)	
Waist–hip measurement				
Low	29 (6.4)	< 0.001	61 (13.5)	0.388
Moderate	40 (12.8)		52 (16.6)	
High	180 (21.5)		134 (16.0)	
Diabetes				
No	204 (13.8)	< 0.001	221 (15.0)	0.119
Yes	45 (34.9)		26 (20.2)	
Cardiovascular diseases				
No	214 (14.1)	< 0.001	235 (15.5)	0.701
Yes	35 (40.7)		12 (14.0)	
Other chronic disease				
No	228 (15.7)	0.611	232 (16.0)	0.058
Yes	21 (14.1)		15 (10.1)	
Family history of hypertension				
No	133 (11.5)	< 0.001	180 (15.6)	0.790
Yes	116 (26.0)		67 (15.0)	
Presence of anxiety symptoms				
No	187 (13.9)	< 0.001	205 (15.2)	0.630
Yes	62 (24.2)		42 (16.4)	
Presence of depression symptoms				
No	176 (13.4)	< 0.001	201 (15.3)	0.791
Yes	73 (25.3)		46 (15.9)	
Total	249 (15.5)		247 (15.4)	

*HTN* hypertension

Moreover, hypertension was particularly prevalent among females (18.6%), individuals with no formal education (20.1%), those currently not employed (19.9%), non-smokers or former smokers (17.4%), those with a high waist-hip ratio (21.5%), individuals with diabetes (34.9%), CVD (40.7%), a family history of hypertension (26.0%), as well as those with symptomatic anxiety (24.2%) and depression (25.3%) (Table 2).

### Best ML model selection

**Fig. 2:**
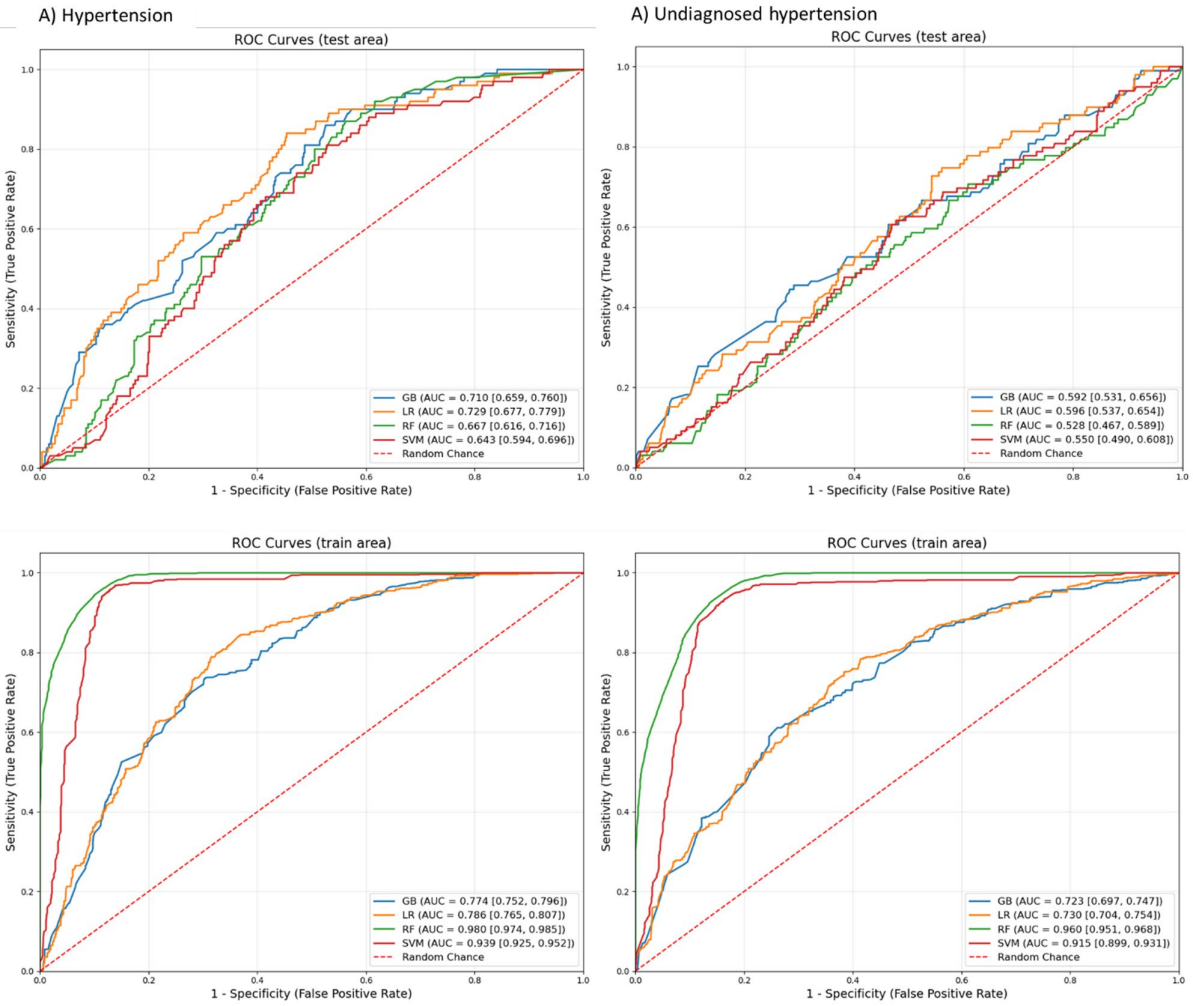
Receiver operating characteristics (ROC) curve in predicting hypertension and undiagnosed hypertension. Note: GB, Gradient Booster; LR, Logistic Regression; RF, Random Forest; SVM, Support Vector Machine

**Fig. 3:**
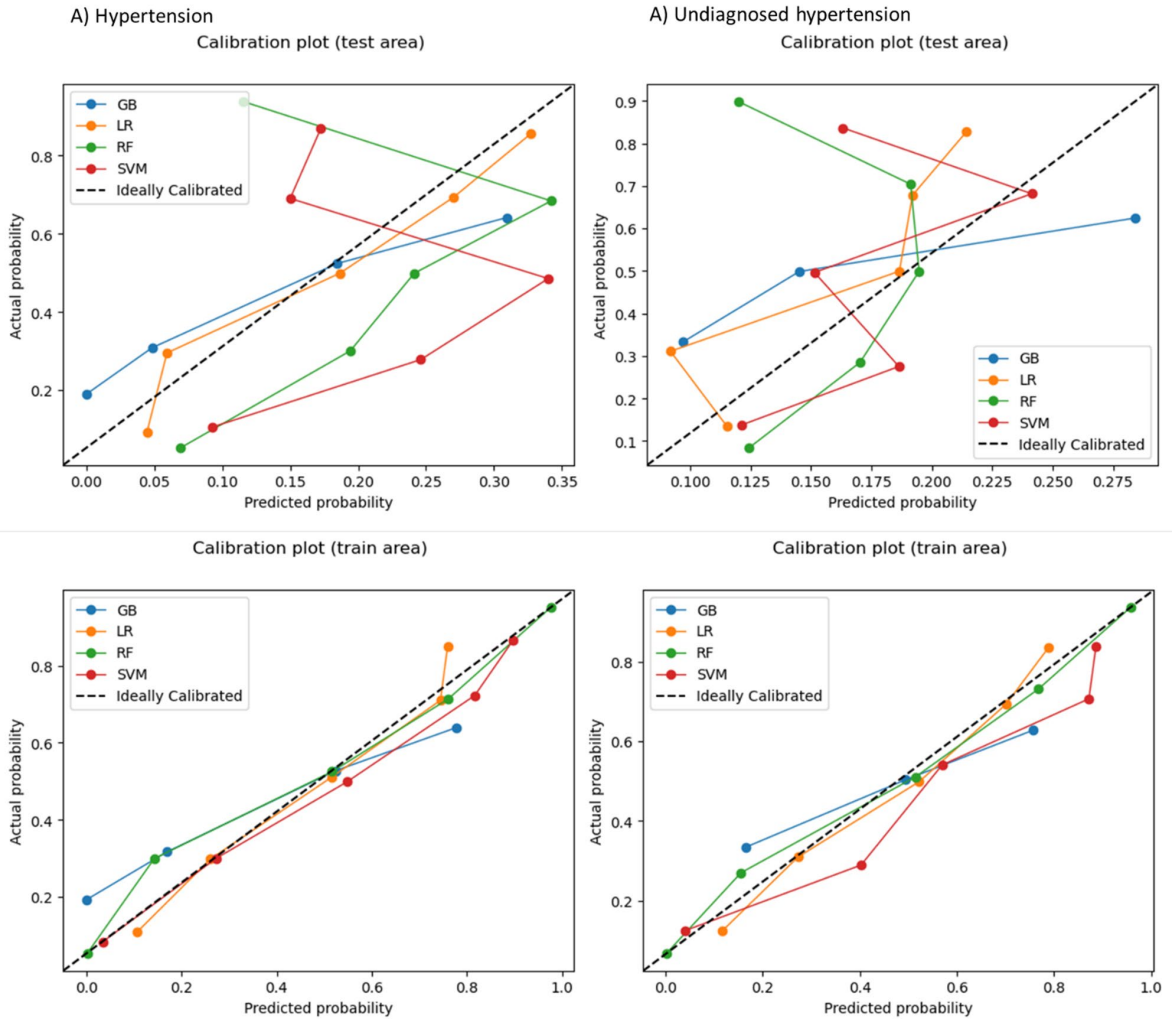
Calibration curve in predicting hypertension and undiagnosed hypertension. Note: GB, Gradient Booster; LR, Logistic Regression; RF, Random Forest; SVM, Support Vector Machine

In predicting hypertension, the LR model outperformed other ML models across most metrics which included precision (0.580), F1 score (0.550) and ROC-AUC score (0.729, 95% CI: 0.677–0.779), as shown in Table 3 and Fig. 2. The calibration curve indicated improved calibration for LR model in predicting hypertension (Fig. 3). The accuracy (0.631), sensitivity (0.690) and specificity (0.620) of LR model were also high.

**Table 3:** Performance metrices of Machine Learning algorithms in predicting hypertension and undiagnosed hypertension

Outcomes	Metrics of algorithms	Algorithms							
		GB		LR		RF		SVM	
		Test	Train	Test	Train	Test	Train	Test	Train
Hypertension	Accuracy	0.582	0.684	0.631	0.736	0.846	0.846	0.712	0.915
	Sensitivity	0.730	0789	0.690	0.800	0.324	0.532	0.230	0.950
	Specificity	0.555	0.580	0.620	0.671	0.806	0.854	0.817	0.878
	Precision	0.580	0.690	0.580	0.740	0.550	0.650	0.520	0.920
	F-1 score	0.520	0.680	0.550	0.730	0.550	0.670	0.529	0.910
Undiagnosed hypertension	Accuracy	0.580	0.667	0.581	0.680	0.629	0.895	0.671	0.884
	Sensitivity	0.566	0.759	0.535	0.716	0.364	0.957	0.273	0.914
	Specificity	0.490	0.572	0.589	0.642	0.678	0.830	0.764	0.852
	Precision	0.520	0.670	0.530	0.680	0.510	0.900	0.510	0.890
	F-1 score	0.450	0.660	0.490	0.680	0.490	0.890	0.510	0.880

*GB* Gradient Booster, *LR* Logistic Regression, *RF* Random Forest, *SVM* Support Vector Machine

In predicting undiagnosed hypertension, the LR model outperformed the other ML models across most evaluation metrics, including a precision of 0.580 and a ROC-AUC score of 0.596 (95% CI: 0.537–0.654), as presented in Table 3 and Fig. 2. The calibration curve (Fig. 3.) also demonstrated that the LR model had better calibration compared to the other ML models for this prediction. The LR model showed moderate accuracy (0.581), sensitivity (0.535), specificity (0.589) and F1 score (0.490) in predicting undiagnosed hypertension. Furthermore, the confusion matrix for all models was presented in the Additional file: Supplementary Fig. 1.

### Influential variables and their interpretability in the RF model

**Fig. 4:**
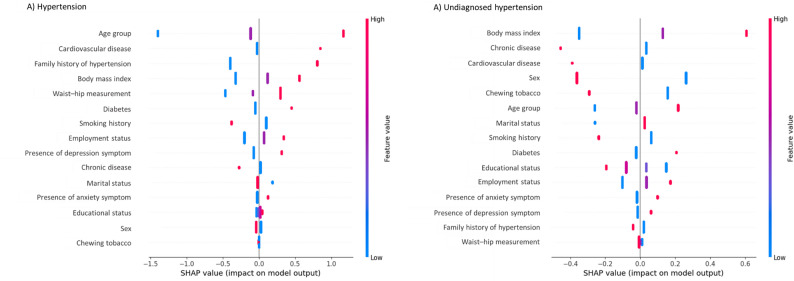
Beeswarm plots for Logistic Regression model to explain the influential factors related to hypertension and undiagnosed hypertension

In (Fig. 4), the SHAP plot provides an overview of the influence of variables, showing the direction of their impact through the distribution of red and blue dots. The vertical bar on the right represents the range of values for the independent variables, with blue indicating low values and red indicating high values. A positive SHAP value for a variable on the x-axis indicates a higher likelihood of the outcome, while a negative value suggests a lower likelihood, such as being normotensive. For instance, the variable “age” is coded as 0 for ≤ 30 years, 1 for 31–50 years and 2 for > 50 years (Additional file: Supplementary Table 1). A value of 0 corresponds to the blue colour, and 2 corresponds to red on the bar. Age emerged as a significant risk factor for hypertension, as the red dots shifted towards positive SHAP values, indicating that individuals who belonged to the age group > 50 years were more likely to develop hypertension. Similarly, factors such as having CVD, having a family history of hypertension, being overweight or obese, having higher waist-hip measurements, having diabetes, being a non-smoker, being retired or a student (currently not employed), having symptoms of depression, and having a chronic disease were associated with hypertension.

Similarly, in (Fig. 4.), influential factors associated with undiagnosed hypertension included being overweight or obese, absence of chronic diseases or CVD, being male, non-use of tobacco, older age (above 50 years), being currently married, non-smoking status, having diabetes, and having no formal education.

## Discussion

There is limited documentation on the status of hypertension and undiagnosed hypertension in rural Bangladesh. Hypertension already diagnosed (15.5%) and undiagnosed (15.4%) hypertension were found to have similar prevalence rates and were notably higher in rural areas. Therefore, the present study explored key factors linked to both hypertension and undiagnosed hypertension among rural Bangladeshi adults, utilising cutting-edge ML algorithms previously recognised as the most effective in predicting hypertension across various populations [21]. Among ML models, the LR outperformed others in predicting hypertension and undiagnosed hypertension. Furthermore, hypertension and undiagnosed hypertension were linked to a range of demographic, behavioural, anthropometric, and clinical factors.

In identifying risk factors of hypertension and undiagnosed hypertension, LR model demonstrated remarkable performance across various metrics, including precision, F1 score, ROC-AUC, and calibration, outperforming other ML models. This aligns with prior research, which also highlighted the potential of the LR method for predicting hypertension, regardless of whether it is diagnosed or undiagnosed [43–45]. In this study, LR outperformed more complex models, such as RF and GB, despite having moderate discriminative ability. This is likely because the dataset was small, contained relatively weak predictors, and modest event rates, conditions under which LR, being a simpler and more controlled model, can perform more reliably. LR is often more robust when the signal-to-noise ratio is modest. It can handle modest signal strength effectively under limited sample sizes. In contrast, RF and GB require larger datasets with stronger signals to capture complex interactions and are more prone to overfitting when the predictive signal is low. As a result, LR provided more stable and generalizable predictions, even though overall performance remained limited. Additionally, studies have identified other algorithms, such as GB, SVM, and RF, as highly effective methods for predicting hypertension [21, 46–49].

The findings suggest that ML models have the potential to provide highly accurate predictions of clinical outcomes and may outperform traditional statistical methods. Integrating ML-based prediction models into rural healthcare in Bangladesh can significantly enhance early detection and resource allocation if embedded into existing workflows rather than creating parallel systems. Community health workers could use mobile apps powered by ML to screen patients during household visits, enabling faster triage and referrals, while mobile health platforms could deliver personalized alerts and reminders to at-risk individuals. Telemedicine services could be strengthened by ML pre-screening that prioritises urgent cases and provides structured risk scores to doctors, improving efficiency in settings with limited physician availability [50]. However, challenges such as incomplete local datasets, infrastructure gaps, digital literacy, and privacy concerns must be addressed through pilot programs, hybrid decision-making that combines AI with human judgment, training for health workers, and government partnerships.

When deploying ML-based health prediction systems in low-resource rural settings like Bangladesh, ethical and privacy considerations become critical to ensure community trust and long-term sustainability. Sensitive health and lifestyle data must be collected, stored, and shared with strict safeguards to prevent misuse or breaches, especially given the limited digital infrastructure and weak regulatory frameworks in rural areas. Transparent communication about how data will be used, coupled with community engagement, can help build trust and reduce fears of surveillance or exploitation. Additionally, ensuring that ML tools are designed with equity in mind, avoiding biases that disadvantage marginalized groups, is essential. By prioritising secure data practices, culturally sensitive implementation, and clear accountability mechanisms, these systems can strengthen healthcare delivery while respecting the rights and dignity of rural populations [50].

In this study, the LR algorithm identified several key factors associated with hypertension, including older age, having CVD, family history of hypertension, overweight or obesity, higher waist-hip measurement, having diabetes, non-smoking status, currently not employed, having symptoms of depression, and chronic disease. Regardless of the use of ML models, factors such as older age, currently not employed, being overweight, non-smoking status and having diabetes consistently emerged as significant contributors to hypertension in rural Bangladesh [5, 33, 51]. Similarly, the LR model selected being overweight or obese, absence of chronic diseases or CVD, being male, non-use of tobacco, older age (above 50 years), being currently married, non-smoking status, presence of diabetes, and having no formal education as influential factors related to undiagnosed hypertension. These findings are consistent with previous studies that primarily employed traditional regression analysis, identifying older age, being male, poor education, being currently married, overweight or obesity, having diabetes and comorbidities as significant risk factors for undiagnosed hypertension [7, 16, 46, 52, 53]. Overall, the risk factors for both hypertension and undiagnosed hypertension identified in this study were found to be important determinants of hypertension in several studies, regardless of their classification [7, 9, 31, 33]. These factors also demonstrated potential for predicting both hypertension and undiagnosed hypertension during ML model development.

The risk factors for hypertension and undiagnosed hypertension in rural Bangladeshi populations differed notably, which may be due to detection bias, health system interaction, health literacy, and personal health behaviour. There were only a few overlapping factors, such as, age over 50, being overweight or obese, having diabetes, and non-smoking status. Greater attention to these risk factors is crucial for better detection and management of both diagnosed and undiagnosed hypertension. Undiagnosed hypertension in rural Bangladeshi populations is influenced by a combination of individual, behavioural, and clinical factors [7, 16, 46, 52, 53]. Being overweight or obese and having diabetes increases the physiological risk of hypertension, yet individuals with these conditions may not seek or receive regular health check-ups [54]. In contrast, those without other illnesses or CVD are less likely to engage with healthcare services, reducing the chances of hypertension being detected [55]. Older adults are more prone to hypertension but may attribute symptoms to normal aging, leading to underdiagnosis [56]. Men, particularly those who are married, often engage less in preventive healthcare due to gender norms and family responsibilities, which lead to the perception that seeking care is unnecessary unless severe symptoms occur [57]. Interestingly, non-smokers and non-tobacco users may be perceived as lower risk by both themselves and healthcare providers, potentially reducing the likelihood of being screened [58]. Furthermore, individuals with no formal education often have limited health literacy and awareness about hypertension and its potential symptoms and risks, which diminishes their likelihood of seeking preventive care [59]. These interconnected factors highlight systemic gaps in awareness, access, and targeted screening efforts, contributing to the persistence of undiagnosed hypertension in this population.

Consistent with the previous finding, approximately 50% of hypertensive patients in rural areas remain unaware of their hypertension status [16]. To address the high burden of undiagnosed hypertension in rural Bangladesh, targeted interventions are needed. These include implementing community-based screening programs, especially for high-risk groups like older adults, men, diabetics, and those with obesity or no formal education. Integrating blood pressure checks into existing chronic disease services and engaging male populations through workplace or market-based outreach can enhance early detection. Health education campaigns are essential for raising awareness among low-literacy populations, while equipping community health workers with training and equipment can improve outreach and referrals [60]. Additionally, mass screening during national health campaigns and strengthening primary healthcare services for routine hypertension monitoring are vital for sustainable control. Collectively, these strategies can bridge awareness, access, and detection gaps to reduce undiagnosed hypertension in rural settings.

This study has several strengths and limitations. Its main strength lies in the successful application of robust machine learning methods and a comprehensive evaluation of factors associated with hypertension and undiagnosed hypertension. However, in limitation, a slightly higher proportion of participants was aged ≥ 50 years than in the national population. Given that hypertension prevalence increases substantially with age, this skewed age structure may have led to an overestimation of the overall prevalence in this study. A locally popular, affordable BP device was used for measurements. To verify its reliability and accuracy, a subset of participants was re-evaluated using a clinically certified BP machine from a nearby clinic, and the readings were compared with those obtained from the survey device at the same time. BP was measured three times, and the average was used to determine hypertension, which could introduce inaccuracies because no variation between measurements was considered. Although taking the average of three readings is standard practice, the prevalence estimates may still be influenced by white-coat effects, as these were not accounted for due to difficulties obtaining clinical records in community-based survey settings. The study was conducted in a single rural area of Bangladesh, which may limit its generalizability due to regional variations in healthcare access, diet, and socioeconomic factors. Caution should be exercised when applying these findings to other rural regions. Consequently, the findings should be interpreted with caution, as the external validity is limited.

Data were collected retrospectively using self-reports, which may have led to underreporting and recall bias. Due to the cross-sectional nature of data, causal relationships could not be assessed. In rural Bangladesh, collecting high-quality clinical and lifestyle data is challenging due to limited health infrastructure, inconsistent record-keeping, low digital literacy, and cultural barriers that affect accurate self-reporting. These gaps often lead to incomplete or unreliable datasets, which in turn weaken the predictive accuracy of ML models. Strengthening data collection through standardized digital tools for community health workers, culturally sensitive survey methods, and integration of mobile platforms for real-time data entry could greatly improve the richness and reliability of health information [61].

Although the LR model identified relevant factors, a ROC-AUC of less than 0.60 for predicting undiagnosed hypertension is generally regarded as non-predictive for screening or clinical decision-making, indicating the model performs only slightly better than chance. Although the LR model performed better than the other ML models, the overall discriminative performance across all models, particularly for undiagnosed hypertension, falls below thresholds typically considered acceptable for clinical prediction.

In real-world contexts, the low ROC-AUC for a model limits its direct applicability as a standalone tool for risk stratification or screening, as its low discrimination may lead to high false-positive and false-negative rates. However, this model can still provide value by highlighting the limitations of available predictors, identifying subgroups, such as older, obese males with low educational attainment, where predictive performance may be stronger, or serving as a baseline for future model development. This study did not compare the performance and findings of the ML-based LR model with those of the traditional LR model to avoid added complexity. Additionally, the ML models used cannot provide p-values or beta coefficients to evaluate associations between variables.

## Conclusion

In Bangladesh, around half of the hypertensive patients were unaware of their status. The LR model demonstrated potential for improved predictions of both hypertension and undiagnosed hypertension and identified influential factors. The risk factors for hypertension and undiagnosed hypertension differed notably, with only a few overlapping factors: age over 50 years, being overweight or obese, having diabetes, and non-smoking status. Other risk factors of undiagnosed hypertension included the absence of chronic diseases or CVD, being male, non-use of tobacco, being currently married, and having no formal education. These findings call for further evaluation of ML applications and risk factors. Moreover, the study highlights the urgent need for stronger national and regional public health initiatives to improve hypertension detection and awareness in rural Bangladesh.

## Supplementary Information

Additional file 1.

## Data Availability

The data that support the findings of this study are available from the corresponding author upon reasonable request.

## Abbreviations

BMI: Body mass index
BP: Blood pressure
CVD: Cardiovascular disease
DBP: Diastolic blood pressure
GB: Gradient Booster
LR: Logistic Regression
ML: Machine Learning
NCDs: Non-communicable diseases
RF: Random Forest
ROC: Receiver Operating Characteristics
SBP: Systolic blood pressure
SHAP: SHapley Additive exPlanations
SVM: Support Vector Machine
