# Supplementary material for: Machine learning approaches in identifying factors associated with hypertension and undiagnosed hypertension in adults in rural areas of Bangladesh
Source: Arch Public Health. 2026 May 18;84:116. doi: 10.1186/s13690-026-01941-z (PMC13185401; doi:10.1186/s13690-026-01941-z)
Supplement: Supplementary file 1 — Additional file 1. [file 13690_2026_1941_MOESM1_ESM.docx]

**S1. Recruitment and training workshop for field personnel**

We have appointed five interviewers (along with two local support persons) with a minimum graduate-level education with social science backgrounds and previous experienced in data collection as ad hoc basis. A registered medical nurse was appointed and trained to measure anthropometrics and blood pressure. The chief investigator primary organized a six-day one week online (Zoom) workshop for the interviewers to familiarise them with this study’s objective and interview questionnaire. All data collectors and support persons strictly followed a safety process (wearing a face mask, maintaining a physical distance of 1.5 meters, and using hand sanitizer before and after each procedure). Additionally, two support persons have been recruited from the local area of the data collection site. They helped the team navigate the area and build rapport with local residents. Mock interviews were conducted as a pre-test to ensure the interviewers were able to practice relevant techniques and adapt to difficult situations during the main interview.

**S2. Data collection instrument**

Data was collected by face-to-face interviews using a validated semi-structured questionnaire. The original questionnaire was in English, which was translated into Bengali for the convenience of local comprehension facilitating data collection. The Bengali version was then converted in to English to ensure linguistic and conceptual equivalency. The survey questionnaire had the following sections: participants’ demographic and life-style factors; and participants’ clinical data, which includes hypertension related questions. As a component of face validity, the research team subjectively evaluated the questionnaires presentation and relevance to ensure the items were pertinent, reasonable, unambiguous, and clear. Additionally, content validity was ensured through a comprehensive literature review, followed by evaluation from an expert panel in the relevant research field.

**S3. Pilot study**

A pilot study was conducted on 24 participants from the selected sampling area to check the acceptability and feasibility of the questionnaire and the average time required to complete the survey. After the pilot study, no major modification was made to the questionnaire except a few minor languages change. The data from pilot study was not included in the study sample. The questionnaire took approximately 30 min to complete per participant.

**S4. Quality of assurance**

To ensure that the quality of data collection was maintained, we have applied several measures: (i) prior the survey, the questionnaire underwent pre-testing to identify and rectify inconsistencies, unclear language, or prolonged administration times. Following appropriate modifications, the questionnaire was finalised; (ii) pre-survey workshop organised for field personnel to outline the purpose of the study, the procedures and potential difficulties related with data collection; (iii) maintained strict monitoring of the data collection and management processes were regularly monitored by one of the local senior investigators; and (iv) utilisation of resilient equipment for physical and clinical measurements. The investigators carried out a random consistency check for at least 5% of the interviewed questionnaires.

**Supplementary Table 1.** Mean of BP measurement

| **BP indicators** | **Mean (95% CI)** |
| --- | --- |
| **SBP** |  |
| 1st measurement | 128.4 (127.5 – 129.3) |
| 2nd measurement | 127.4 (126.5 – 128.3) |
| 3rd measurement | 126.7 (125.9 – 127.6) |
| **DBP** |  |
| 1st measurement | 80.6 (80.1 – 81.1) |
| 2nd measurement | 80.3 (79.7 – 80-8) |
| 3rd measurement | 79.9 (79.4 – 80.4) |

**Note:** BP, blood pressure; SBP, systolic blood pressure; DBP, diastolic blood pressure; CI, confidence interval.

**Supplementary Table 2.** Measurements of independent variables

| **Variable** | **Description** | **Measurement (coding)** | **Scale of measurement** |
| --- | --- | --- | --- |
| **Demographic variables** | | | |
| Age | Participants age in years during the data collection period | <35 (0), 35-50 (1) and ≥51 (2) | Categorial |
| Sex | Sex of the participants | Male (0), female (1) | Binary |
| Educational status | Educational status can be categorised as "educated" when they have completed a minimum of five years of schooling, typically corresponding to the primary (class 1 to 5), secondary (class 6 to 10), and/or higher level. On the other hand, if there are no schooling years completed (0 years of schooling), this categorised them as "no formal education." | No formal education (0), primary (1), secondary (2), or higher (3) | Categorical |
| Employment status | Participants engaged in economic activity at the time of data collection. | Employed or self-employed (0), housewife (1), retired or student (2) | Categorical |
| Marital status | Participant’s marital relationship status during the data collection period. | Never married, separated, divorced, or widowed) (0), currently married (0) | Binary |
| **Lifestyle factors** | | | |
| Chewing tobacco | Adaptable behaviours and ways of life like influence the participant’s health and well-being. | Past and non-user (0), current user (1) | Binary |
| Smoking history | Adaptable behaviours and ways of life like influence the participant’s health and well-being. | Past and non-smoker (0), current smoker (1) | Binary |
| **Anthropometric data** | | | |
| Body mass index (BMI) | BMI was derived from the measured values of height and weight. Using WHO’s BMI guidelines for the Asian population, BMI has been classified. **Height** was measured using a portable stadiometer (Seca 213 Portable Stadiometer) with light clothing and while barefoot. Study participants were instructed to stand straight against a wall looking forward with loosely hanging arms on their sides. The head plate was put on the crown of the head and a measurement on tape was noted to the nearest 0.5 cm. The measurement was taken twice. If the difference was more than 2 mm, a third measurement was taken. The average of the measurements was recorded as the participant's height.  A third measurement was taken if the difference was more than 2 mm. The average of the measurements was recorded as the participant’s height. Further, participants’ **weight** was measured using a digital weighing scale (BEURER wellbeing PS 240*)* with light clothing and while barefoot. Participants were instructed to stand still in the center of the machine looking forward with loosely hanging arms on their sides. Weight was recorded to the nearest 0.1 kg. | Underweight (<18.50 kg/m^2^) and normal (18.50-22.99 kg/m^2^) (0), overweight (23.00-27.49 kg/m^2^) (1), and obese (≥27.50 kg/m^2^) (2) | Categorical |
| Waist–hip measurement | The waist–hip ratio is the dimensionless ratio of the circumference of the waist to that of the hips. This was calculated as waist circumference (WC) divided by hip circumference (HC) (WC⁄HC). Optimal cut-off values of waist–hip ratio was measured. The cut-off points are 85 cm for Asian men and 75–80 cm for Asian women and categorised as low, moderate and high. Participants’ WC was measured while they were wearing thin clothing (if they were clothed for cultural reasons), on exhalation, midway between the lower rib margin and the anterior superior iliac spine (hip bone), or the narrowest abdominal point. Participants were relaxed with arms hanging loosely by their sides. The tape measure was kept horizontal for a standing measurement. This was done twice. If the measurements differed by more than 2 cm, a third measurement has been taken. The average of the measurements was recorded as the participant’s WC. Further, a measurement was taken at the widest circumference around the hip bones so that the tape passes over the greatest protrusion of the gluteal muscles. The tape measure was kept horizontal for a standing measurement. This was done twice. If the measurements differed by more than 2 cm, a third measurement was taken. The hip circumference was recorded to the nearest 0.5 cm. | Low (0), moderate (1) and high (2) | Categorical |
| **Clinical characteristics** | | | |
| Diabetes | Participant living with diabetes mellitus was considered if there was a documented diagnosis and/or medication history. | No (0), yes (1) | Binary |
| Cardiovascular diseases | Patients had heart attack and stroke received treatment and/or were taking prescribed medication. | No (0), yes (1) | Binary |
| Other chronic disease | Chronic diseases such as kidney disease, asthma, cancer, arthritis or others were considered to be present if there was a documented diagnosis, medication history, or any past procedures. | No (0), yes (1) | Binary |
| Family history of hypertension | Any blooded family member had high blood pressure | No (0), yes (1) | Binary |
| Presence of anxiety symptoms | The Generalized Anxiety Disorder-7 (GAD-7) scale comprises seven questions. The scale GAD-7 showed high internal consistency with Cronbach's α being 0.78. Participants were asked to have anxiety symptoms in two weeks prior to the interview a 4-point Likert scale with options 0 (not at all), 1 (several days), 2 (more than half the days), and 3 (nearly every day). Based on the different cut-off of GAD-7 score, anxiety was categorized as no or minimal anxiety (0-4), mild anxiety (5-9), moderate anxiety (10-14), and severe anxiety (≥15). It was further coded binary as ≤4 for no or minimal anxiety and 1 coded for mild to severe anxiety symptom for rest of the score (score: >4). | No (0), yes (1) | Binary |
| Presence of depression symptoms | Depression symptom was assessed using the Patient Health Questionnaire-9 (PHQ-9) scale which consists of nine questions. The PHQ-9 scale demonstrated strong internal consistency, with a Cronbach's α of 0.73. Participants were asked to have depressive symptoms in two weeks prior to the interview using a 4-point Likert scale with options 0 (not at all), 1 (several days), 2 (more than half the days), and 3 (nearly every day). Adding up scores for individual question makes a total score of 27. Based on the different cut-off of PHQ-9 score, symptomatic depression was categorized as no or minimal depression (0-4), mild depression (5-9), moderate depression (10-14), moderately severe depression (15-19) and severe depression (20-27). A cutoff score of 4 was used to determine the mild to severe presence of depression symptom. | No (0), yes (1) | Binary |

**Supplementary Table 3.** Model optimization

| **Items** | **Hyperparameter optimization** | **Cross-validation (k-fold)** | **Parameters** | **Thresholds** |
| --- | --- | --- | --- | --- |
| Gradian Booster (GB) | Grid search | 5 folds | n_estimators, min_samples_split,  min_sample_leaf,  learning_rate, learning_rate, max_features, sub_smaple, scoring, n_jobs, random_state | 0.5 |
| Logistic Regression (LR) | Grid search | 5 folds | random_state, C, penalty, solver, scoring | 0.5 |
| Random Forest (RF) | Grid search | 5 folds | n_estimators, min_samples_split,  max_features | 0.5 |
| Support Vector Machine (SVM) | Grid search | 5 folds | kernel, probability, 'C', gamma | 0.5 |

**Supplementary Table 4.** Defining performance metrics

| True positive | A true positive is an outcome where the model correctly predicts the positive class |
| --- | --- |
| True negative | A true negative is an outcome where the model correctly predicts the negative class |
| False positive | A false positive is an outcome where the model incorrectly predicts the positive class |
| False negative | a false negative is an outcome where the model incorrectly predicts the negative class. |
| Accuracy | Model accuracy is defined as the number of classifications a model correctly predicts divided by the total number of predictions made which can be represented as: Accuracy = (True positives + True negatives) / (True positives + True negatives + False positives + False negatives). |
| Sensitivity/recall | Sensitivity refers to a test's ability to designate an individual with disease as positive. A highly sensitive test means that there are few false negative results, and thus fewer cases of disease are missed. Sensitivity = true positive /(false negative + true positive) |
| Specificity | Specificity measures the proportion of true negatives that are correctly identified by the model. Specificity = true negative / (true negative + false positive) |
| Precision | The formula for precision (also called positive predictive value, PPV) is:  Precision = True positive/(True positive + False positive)  It measures the proportion of correctly predicted positive cases out of all predicted positive cases |
| F1 score | The **F1 score** is the harmonic mean of **precision** and **recall,** balancing both metrics. It is calculated as:  F1=2*(Precision*recall)/(Precision + Recall)  The F1 score provides a balance between precision and recall, making it useful when false positives and false negatives have similar consequences.  Interpretation:   - F1 = 1 → Perfect precision and recall (ideal model). - F1 = 0 → The model has either zero precision or zero recall (worst performance). - Higher F1 Score → Better model performance, meaning it effectively identifies positive cases while minimizing false positives and false negatives. - Lower F1 Score → Poor performance, indicating the model struggles with either false positives or false negatives.   The F1 score is crucial for imbalanced datasets, where accuracy alone can be misleading. |
| Receiver Operating Characteristics (ROC) | The plot of sensitivity versus 1-Specifity is called receiver operating characteristic (ROC) curve and the area under the curve (AUC). The mathematical formula of AUC is as follows  ROC*=*$\int_{x=0}^{1} [Sensitivity \{\left( 1-Specificity \right)^{-1} \left( x \right)\}]dx$ |
| Calibration plot | A calibration plot visualizes the alignment between predicted probabilities and actual outcomes, with the x-axis representing predicted probabilities and the y-axis showing observed event proportions. A diagonal line indicates perfect calibration, while deviations from this line reveal miscalibration: predictions above the line suggest underestimation, and those below the line indicate overestimation. |


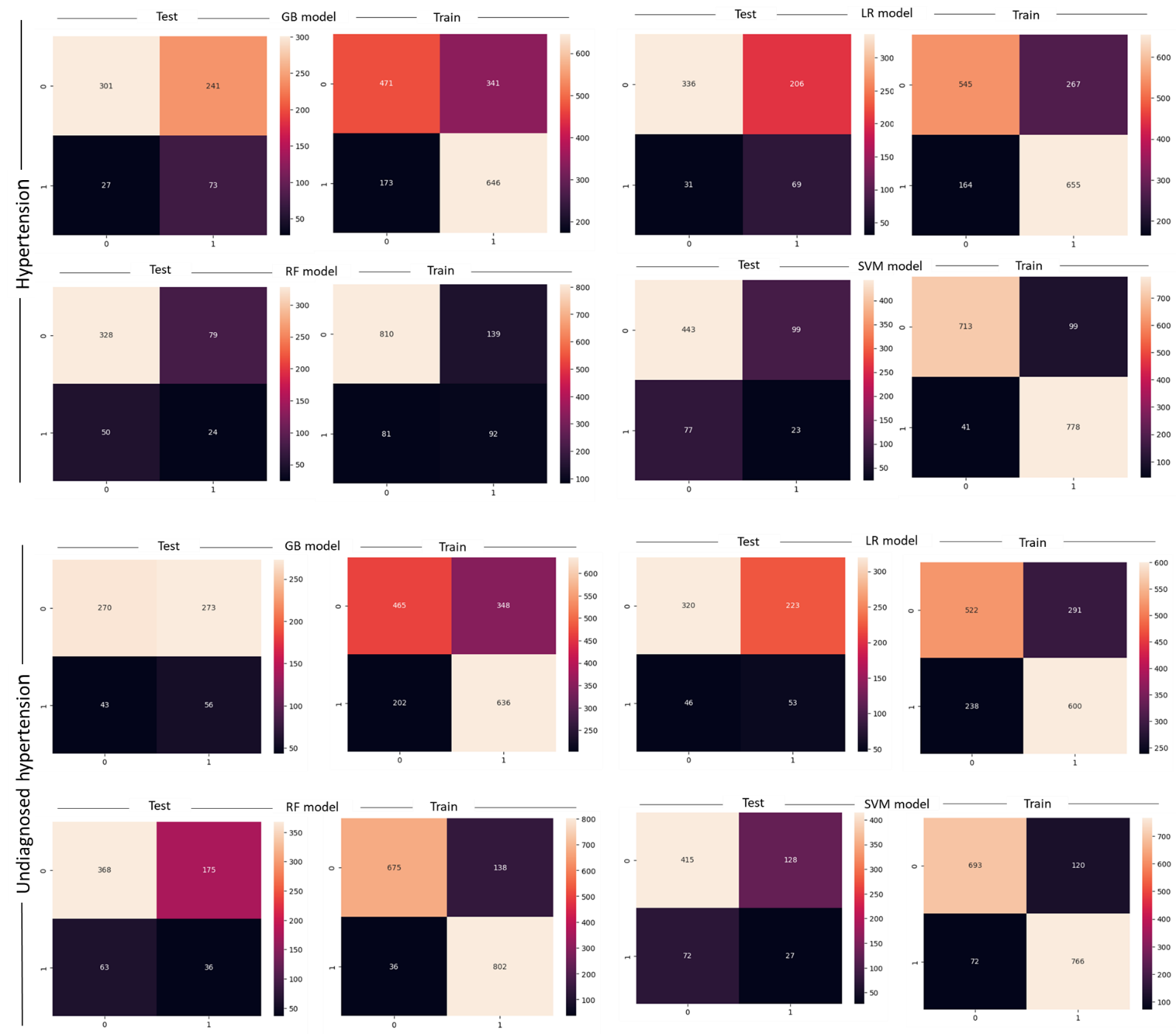


**Supplementary** **Fig. 1.** Confusion matrix
